# Actionable Genomic Alterations and Survival in Gallbladder Cancer: A Documented Stage- and Treatment-Matched Real-World Global Analysis

**DOI:** 10.3390/cancers18091452

**Published:** 2026-05-01

**Authors:** Zeeshan Solangi, Katherin Zambrano-Vera, Laura Haas, Antonio J. Arciniegas, Zina Agha, Ghulam Shah, Ahmed Abbasi, Werner Kristjanpoller, Olga Kozyreva, Fernando Rotellar, Eduardo A. Vega

**Affiliations:** 1 Department of Internal Medicine, Boston Medical Center South, Brockton, MA 02301, USA; 2 Department of Internal Medicine, Berkshire Medical Center, Pittsfield, MA 01201, USA; kzambrano@bhs1.org (K.Z.-V.);; 3 Department of Surgery, Boston Medical Center Brighton, Boston, MA 02135, USA; laura.haas@bmc.org; 4 Department of Medicine, Brigham and Women’s Hospital, Boston, MA 02115, USA; 5 College of Medicine, Hawler Medical University, Erbil 44001, Iraq; 6 Department of Medicine, NYU Grossman School of Medicine, New York, NY 10016, USA; 7 Department of Industries, Universidad Tecnica Federico Santa Maria, Valparaiso 2390123, Chile; werner.kristjanpoller@usm.cl; 8 Department of Oncology, Dana Farber at Boston Medical Center Brighton, Boston, MA 02135, USA; 9 Department of Surgery, Clinica Universidad de Navarra, 31008 Pamplona, Spain

**Keywords:** gallbladder cancer, *ERBB2*, *KRAS*, real-world evidence, genomics, survival, TriNetX

## Abstract

Background: GBC is an aggressive biliary tract malignancy with limited survival. Although actionable genomic alterations (AGAs) are increasingly recognized in GBC, their prognostic association in real-world practice remains incompletely defined because genomic status is often confounded by stage at presentation and treatment selection. We evaluated the association between documented AGA status and overall survival (OS) using a tiered matching strategy to account for major clinical confounders. Methods: Using the TriNetX Global Collaborative Network, we identified adults with GBC and stratified them into patients with at least one documented AGA in *KRAS*, *TP53*, *ERBB2*, *IDH1*, *FGFR1*, *PIK3CA*, or *ARID1A*, and a comparison cohort with no documented AGA (representing a real-world population of untested and wild-type patients). Two 1:1 propensity score-matched models were constructed: Model 1 matched for age, sex, and race/ethnicity; Model 2 additionally matched for metastatic disease, surgical resection, and chemotherapy history. Outcomes were evaluated using risk analysis and Kaplan–Meier survival methods. Results: A marked disparity in genomic documentation was observed before matching, with unknown race recorded in 51.0% of the comparison cohort versus 3.7% of the documented AGA cohort. In the demographic-matched analysis (Model 1), the documented AGA cohort had higher mortality (52.8% vs. 42.5%, *p* < 0.001) and shorter median OS (684 vs. 948 days; HR 1.23, *p* = 0.006). In the primary stage- and treatment-matched analysis (Model 2), mortality remained higher in the documented AGA cohort (56.2% vs. 43.0%), corresponding to an absolute risk difference of 13.2% (*p* < 0.001). Median OS was numerically shorter in the documented AGA cohort (750 vs. 784 days), although the proportional hazards assumption was violated, supporting interpretation based primarily on absolute risk measures. In exploratory subgroup analyses, *KRAS* alterations were associated with worse survival, whereas the *TP53* subgroup was limited by small sample size. Conclusions: In this real-world matched analysis, the presence of documented AGAs in GBC were associated with higher mortality even after matching for major demographic, stage-related, and treatment-related variables. These findings support the prognostic relevance of genomic status in GBC and underscore the need to address disparities in access to genomic documentation and testing.

## Simple Summary

Gallbladder cancer (GBC) is a highly aggressive disease with limited survival. It remains unclear whether actionable genomic alterations (AGAs) independently affect outcomes beyond disease stage and treatment. In this global real-world analysis of over 40,000 patients with GBC, we compared cohorts with and without a documented AGA matched for demographic, stage-related, and treatment-related factors. Patients with a documented AGA had a significantly higher risk of death (56.2% vs. 43.0%), representing a 13% absolute increase, suggesting that tumor biology plays a key role in long-term outcomes. We also found major disparities in who receives genomic testing. These results highlight the prognostic importance of genetic testing and the need to expand equitable access to genomic profiling in GBC.

## 1. Introduction

GBC is the most common malignancy of the biliary tract [1]. Despite its relatively low incidence in Western populations, it remains highly lethal, characterized by a high burden of mortality and a five-year survival rate that has seen little improvement over decades [1]. Most patients remain asymptomatic during early disease. Consequently, diagnosis often occurs at advanced stages, requiring aggressive multidisciplinary care [2]. While clinical staging and surgical resectability have historically been the primary determinants of prognosis [3,4,5], the emergence of precision oncology, including HER2-targeted therapies [6] and PI3K pathway inhibitors, has shifted focus toward the underlying molecular drivers of the disease [7].

Recent advances in molecular profiling have begun to characterize the genomic landscape of GBC [8,9]. Recurrent alterations in oncogenic drivers, including *KRAS*, *ERBB2*, *PIK3CA*, *ARID1A*, *FGFR* family genes, *IDH1*, and *TP53*, have been identified as key contributors to tumorigenesis and potential therapeutic targets [10,11,12]. However, translating these genomic findings into reliable prognostic insights has proven challenging. Most existing studies rely on small, single-institution cohorts or narrow sequencing panels, often failing to account for the powerful confounding effects of clinical stage and treatment intensity [13]. Consequently, it remains unclear whether these genomic alterations contribute to mortality independently of traditional clinical risk factors or merely reflect a higher burden of advanced disease at presentation.

Because GBC incidence is often associated with specific geographic and ethnic clusters, a large-scale analysis of a diverse, real-world population presents a unique opportunity to separate biological drivers from clinical confounders. In this study, we used the TriNetX Global Collaborative Network to evaluate mortality and survival outcomes in GBC, stratified by the presence of AGAs. While the use of large-scale electronic health record (EHR) data provides unprecedented statistical power, we acknowledge that these real-world datasets often lack the granular surgical and oncologic detail found in prospective registries, specifically, precise data on surgical margin status (R0 vs. R1), lymph node yield, and the specifics of multi-agent chemotherapy regimens.

To ensure a rigorous assessment of biological risk, despite these inherent data limitations, we employed a tiered statistical approach: first, establishing a real-world baseline through demographic matching, and second, isolating the independent prognostic impact of AGAs through matching for metastatic status, surgical intervention, and chemotherapy history. Our goal was to quantify the independent lethality of these genomic drivers and identify disparities in testing access, thereby informing more equitable and precise molecular risk stratification in GBC.

## 2. Methods

### 2.1. Data Sources and Study Population

We conducted a retrospective, real-world evidence analysis using the TriNetX Global Collaborative Network (Cambridge, MA, USA) [14], a federated global research network comprising de-identified electronic health records (EHRs) data from 157 to 166 healthcare organizations. Data were obtained via queries executed on 18 November and 10 December 2025.

The study population was restricted to adults ≥ 18 years with a documented diagnosis of GBC, defined by ICD-10-CM code C23 or ICD-O-3 code C23. To minimize historical bias from the pre-genomic era and ensure comparability within the contemporary era of molecular testing, we restricted the study population to patients whose index GBC diagnosis occurred on or after 1 January 2015. The earliest recorded GBC diagnosis within this window served as the index event. Outcomes were analyzed beginning one day after the index event and continued throughout the patient’s subsequent record to mitigate immortal time bias.

### 2.2. Selection of Genes and Cohort Definitions

Patients were stratified into two cohorts based on the presence of AGAs commonly implicated in biliary cancers. To maintain methodological transparency and acknowledge the inherent selection biases in real-world sequencing data, we defined our cohorts as follows:

Cohort 1 (no documented AGA): Included patients with GBC who had no documented alterations in the selected gene panel within their electronic health record (EHR). Crucially, as this cohort is derived from real-world clinical documentation, it likely contains a mixture of patients who are biologically wild-type and those who never underwent genomic profiling. Consequently, the comparison represents “documented AGA-positive” versus “not documented/untested” status, rather than a definitive comparison of mutated versus wild-type biology.

Cohort 2 (≥1 documented AGA): Included patients with GBC and at least one documented alteration in *KRAS*, *TP53*, *ERBB2*, *IDH1*, *FGFR1*, *PIK3CA*, or *ARID1A*.

Genomic status was identified using global oncology codes (TNM [Tumor, Node, Metastasis staging system], UMLS [Unified Medical Language System]), procedure codes (CPT [Current Procedural Terminology]), and genomic entries (GENE [gene-level results]) within the EHR data.

### 2.3. Two-Tiered Propensity Score Matching

**Figure 1:**
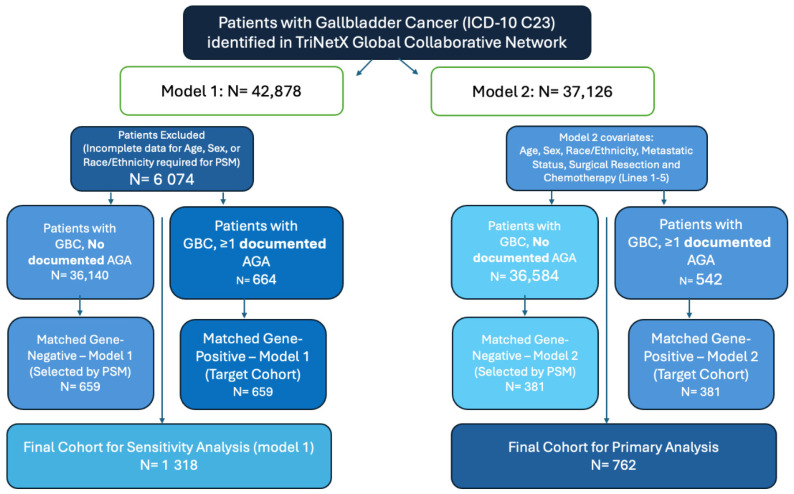
CONSORT Diagram illustrating study population selection and the two-tiered propensity score matching (PSM) strategy. Patients with gallbladder cancer (GBC) were identified in the TriNetX Global Collaborative Network. Model 1 (Sensitivity Analysis): Balanced 1:1 for age, sex, and race/ethnicity, resulting in a final cohort of *N* = 1318 (*N* = 659 per group). Model 2 (Primary Analysis): Balanced 1:1 for demographic variables plus metastatic status, surgical resection, and chemotherapy history, resulting in a final cohort of *N* = 762 (*N* = 381 per group). Cohort Definitions: The diagram details the exclusion of 6074 patients due to incomplete demographic data and the stratification into cohorts with and without documented actionable genomic alterations (AGAs). Subgroup Distribution: Before matching, documented AGAs included *KRAS* (*N* = 563), *TP53* (*N* = 34), *ERBB2* (*N* = 24), and others (*PIK3CA, ARID1A, IDH1, FGFR1*; each *N* = 10).

To isolate the independent prognostic impact of documented AGAs from clinical and demographic confounders, we performed 1:1 nearest-neighbor propensity score matching without replacement using a two-model tiered approach (Figure 1).

We acknowledge that while Model 2 accounts for major clinical interventions, these variables serve as broad proxies for treatment intensity. Specifically, the TriNetX database does not provide granular details on surgical quality (e.g., R0 vs. R1 resection status or lymph node yield) or the specific composition of chemotherapy regimens.

Model 1 (demographic/sensitivity analysis): Balanced the cohorts for age, sex, and race/ethnicity (including White, Black or African American, Hispanic or Latino, and Unknown categories). From a starting population of *N* = 42,878, this resulted in two balanced cohorts of *N* = 659 patients each (SMD ≤ 0.034).

Model 2 (oncologic/primary analysis): This model expanded upon the demographic covariates of Model 1 to include clinical markers of disease severity and treatment intensity to further isolate the impact of genomic documentation. These variables included metastatic status at index (ICD-10 C76–C80), surgical resection of the gallbladder or biliary tract, and history of systemic chemotherapy. From a refined cohort of *N* = 37,126 patients with complete oncologic records, 1:1 matching generated two balanced groups of *N* = 381 patients each were generated (SMD ≤ 0.048).

Covariate balance across all matched variables was assessed using standardized mean differences (SMD), with a threshold of ≤0.1 utilized to define a successful balance between cohorts.

### 2.4. Study Outcomes and Statistical Analysis

The primary outcome was all-cause mortality, defined using the TriNetX “deceased” term. All analyses were performed within the TriNetX built-in analytics platform. For comparative risk analyses, cumulative risk, risk ratio (RR), risk difference (RD), and odds ratio (OR) were calculated, with statistical significance assessed using a two-sided z-test. Time-to-event outcomes were analyzed using the Kaplan–Meier method and compared with the log-rank test. The proportional hazards assumption was evaluated using the Grambsch–Therneau method [15]. When this assumption was violated (*p* < 0.05), absolute risk difference and cumulative mortality risk were prioritized over the hazard ratio (HR) to interpret effect size, as these measures more accurately reflect outcomes when the hazard is non-constant over time. The forest plot summarizing subgroup hazard ratios was generated using RStudio (Version 2026.01.0+392; Posit Software, PBC, Boston, MA, USA).

To address the biological heterogeneity inherent in the broad AGA-positive cohort, exploratory subgroup survival analyses were performed within the matched Model 2 cohort for the most prevalent individual alterations (*KRAS, TP53* and *ERBB2*). As these subgroup comparisons represent a secondary analysis of a matched population, they were interpreted as hypothesis-generating and were not adjusted for multiple comparisons.

## 3. Results

### 3.1. Genomic Testing Disparities and Selection Bias

A total of 42,878 patients were identified for the initial analysis. Evaluation of the pre-matched cohorts revealed a profound disparity in genomic documentation. A total of 51.0% of patients in the cohort without a documented AGA had an unknown race classification, compared to only 3.7% in the cohort with a documented AGA (*p* < 0.001). Among patients with documented race, White patients demonstrated a significantly higher prevalence of genomic results (67.1% vs. 32.6%, *p* < 0.001). These findings suggest a systemic selection bias in real-world genomic profiling, where molecular testing is more frequently documented for patients with higher-quality demographic data.

### 3.2. Baseline Characteristics and Propensity Score Matching

Propensity score matching was successfully executed at two levels of clinical rigor. As shown in Table 1, Model 2 achieved a high-fidelity balance for clinical markers of disease severity and treatment intensity that were not included in Model 1 (Table 1).

### 3.3. All-Cause Mortality Risk Analysis

The association between documented AGAs and increased mortality remained robust across both matching strategies:

Model 1 (Sensitivity Analysis): All-cause mortality was significantly higher in the cohort with a documented AGA (52.8%) compared to those without (42.5%). Representing a 10.3% absolute risk increase (*p* < 0.001).

Model 2 (Primary Analysis): Despite adjusting for stage and treatment, the documented AGA cohort maintained a higher mortality risk (56.2%) than the comparison group (43.0%). This corresponds to an absolute risk increase of 13.1% (Risk Difference [RD] = 0.132, *p* < 0.001) (Table 2.).

### 3.4. Kaplan–Meier Survival Analysis

**Figure 2:**
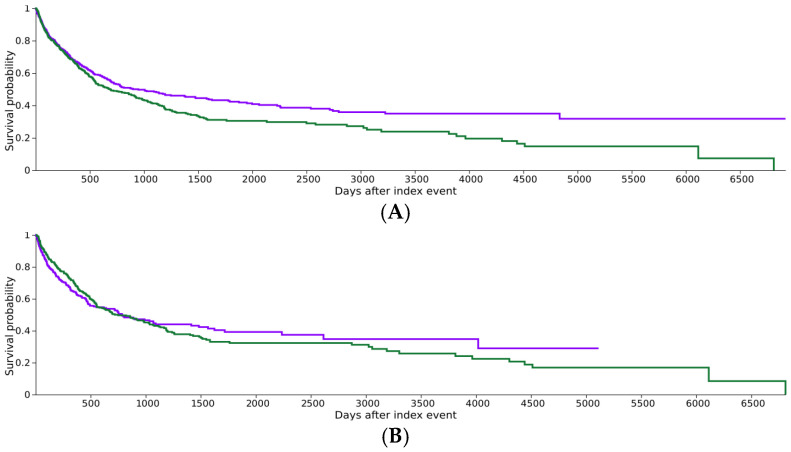
Kaplan–Meier Survival. (**A**) Overall survival of GBC patients with documented AGA versus no documented AGA in the demographic-matched cohort (Model 1). (**B**) Overall survival in the stage- and treatment-matched cohort (Model 2). Purple line: Cohort 1 (no documented AGA). Green line: Cohort 2 (documented AGA). Note: In Model 2, the curves exhibit significant overlap (log-rank *p* = 0.925); however, due to the violation of proportional hazards (*p* = 0.002), the study primary findings are based on the absolute risk difference in cumulative mortality.

Time-to-event results varied according to the degree of clinical matching. In the demographic-matched analysis (Model 1), the documented AGA cohort exhibited significantly shorter median OS than the cohort without a documented AGA (684 vs. 948 days; HR 1.23, *p* = 0.006; Figure 2A). In the primary stage- and treatment-matched analysis (Model 2), median OS was comparable between the documented AGA cohort and the comparison cohort (750 vs. 784 days; log-rank *p* = 0.925; Figure 2B). The log-rank test was not significant, but the proportional hazards assumption was violated (χ^2^ = 9.979, *p* = 0.002). This indicates a time-varying effect. Accordingly, absolute mortality risk and risk difference were used as the primary effect measures for Model 2. While the survival curves in Figure 2B show significant overlap throughout much of the follow-up period, the final cumulative mortality was substantially higher in the documented AGA group (56.2% vs. 43.0%). This suggests that while AGAs may not influence the immediate timing of mortality after clinical intervention, they are associated with a higher overall risk of death by the end of the study period.

### 3.5. Hypothesis-Generating Subgroup Observations

To explore the potential biological heterogeneity within the pooled AGA cohort, exploratory subgroup analyses were performed for the most prevalent alterations. The clinical and demographic distribution of these specific subgroups, including distributions of age, sex, and race, is detailed in Supplementary Table S1. These analyses are hypothesis-generating and are subject to the same “no documented AGA” comparator limitations as the primary analysis.

Among these, *KRAS* alterations (*N* = 563) demonstrated a significant adverse survival signal, with a median survival of 673 days compared to 1331 days in the matched cohort without a documented *KRAS* alteration (HR 1.279, 95% CI 1.074–1.524; *p* = 0.004). In contrast, neither *TP53* (*N* = 33; HR 0.635, 95% CI 0.308–1.309; *p* = 0.255) nor *ERBB2* (*N* = 17) alterations were associated with statistically significant survival differences in this dataset. Other documented alterations, including *PIK3CA, ARID1A, IDH1*, and *FGFR1* (each *N* = 10), occurred with insufficient frequency to permit individual comparative survival analyses.

**Figure 3:**
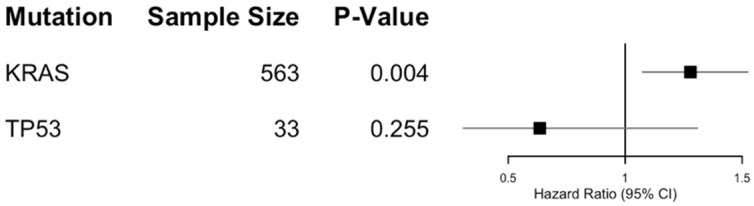
Forest Plot of Mortality Risk by Specific Genomic Alterations. Data represent Hazard Ratios (HR) and 95% Confidence Intervals (CI) for all-cause mortality within the Model 2 matched cohort. Subgroups include *KRAS* and *TP53* alterations compared to the cohort with no documented alterations for each respective gene.

These results suggest that the adverse prognostic signal observed in the primary analysis may be largely driven by the *KRAS*-mutated subgroup. However, given the potential for statistical artifacts when performing subgroup analyses on a previously matched population, and the limited power to detect differences in the smaller non-*KRAS* subgroups, these findings should be interpreted with caution. (Figure 3).

## 4. Discussion

In this large, real-world analysis of GBC patients, documented AGAs were associated with higher mortality, even after adjusting for key demographic, stage, and treatment factors. This association remained robust after matching for major demographic, stage, and treatment variables.

A key challenge in GBC research is distinguishing whether genomic alterations simply reflect more advanced disease at presentation or remain associated with outcomes after accounting for clinical severity. To address this, our primary model incorporated matching for metastatic disease, surgical resection, and chemotherapy history. In that more restrictive comparison, the documented AGA cohort continued to demonstrate a higher absolute mortality risk than the comparison cohort without a documented AGA. This pattern supports the prognostic relevance of genomic alterations in GBC [16,17], However, it is important to note that our comparison cohort represents “no documented AGA status”, which likely includes both biologically wild-type patients and those who never underwent molecular profiling.

Interestingly, the absolute risk difference increased from 10.3% in Model 1 to 13.2% in Model 2. While adjusting for clinical confounders typically attenuates effect sizes, this divergence suggests that the prognostic impact of documented AGAs becomes more pronounced when comparing patients with similar treatment histories and disease stages. This may indicate that AGAs identify a subset of patients who derive less benefit from standard-of-care interventions, such as systemic chemotherapy or surgical resection. This is a challenge recently contextualized by Yoon and Yoo (2026) [18] in their assessment of current therapeutic ceilings in biliary tract cancers. Alternatively, this shift may reflect a “documentation bias”, where patients with more aggressive clinical courses are more likely to undergo comprehensive genomic profiling and have complete oncologic data recorded in the EHR.

The observed increase in mortality among patients with AGAs likely reflects the biological impact of oncogenic driver pathways that promote tumor progression, treatment resistance, and metastatic potential. In particular, alterations in *KRAS* and related signaling networks may lead to sustained activation of the RAS/MAPK pathway, contributing to uncontrolled proliferation and reduced sensitivity to systemic therapies. Similarly, co-occurring alterations in *PI3K*/*AKT* and receptor tyrosine kinases, including *ERBB2*, may further enhance tumor aggressiveness through redundant signaling mechanisms. These overlapping molecular processes may help explain why survival differences persist even after controlling for stage and treatment, suggesting that genomic alterations are not merely markers of advanced disease but active determinants of tumor behavior, though the magnitude of their impact may be influenced by the clinical context of testing access. The clinical relevance of these specific drivers in our cohort, compared to frequencies and prognostic markers reported in existing genomic literature, is summarized in Table 3.

The survival analysis in Model 2 also demonstrated violation of the proportional hazard assumptions, suggesting that the association between documented AGA status and mortality may vary over time. Rather than relying primarily on the hazard ratio in this setting, we emphasized absolute mortality risk and risk difference, which provide a more stable summary of effect when hazards are non-proportional. The significant overlap of the survival curves in the first 12–18 months of Model 2 (Figure 2B) likely reflects the immediate prognostic benefit of surgical resection and systemic chemotherapy, which were balanced between cohorts. However, as the impact of these initial intervention plateaus, the underlying biological aggression associated with documented AGAs appears to drive the divergence in final cumulative mortality. This interpretation suggests that clinical interventions may attenuate early survival differences, whereas underlying tumor biology plays a more substantial role in longer-term outcomes; however, this interpretation should be considered hypothesis-generating.

The prognostic significance of documented AGAs in our cohort is further contextualized by current genomic frameworks and clinical benchmarks (Table 3). Our observation that *KRAS* serves as a primary driver of adverse outcomes is reinforced by Guangchao et al. (2026) [19], who recently highlighted the synergistic mortality risk associated with concurrent *TP53* and *KRAS* alterations—a combination that frequently appeared in our high-mortality subgroups. These molecular drivers are increasingly recognized as central to the “standard of care” for advanced biliary tract cancers, as detailed by Yoon and Yoo (2026) [18] and Canale et al. (2021) [7], who emphasize that identifying targetable alterations like *ERBB2* and *PIK3CA*—extensively characterized by Giraldo et al. (2022) [16]—is essential for optimizing therapeutic sequencing. Furthermore, the demographic documentation gaps and testing disparities identified in our analysis mirror the broader inequities described by Abboud et al. (2024) [20] and Heath et al. (2024) [21], whose findings suggest that racial and ethnic disparities in molecular testing documentation continue to hinder the equitable implementation of precision oncology. Finally, the capacity for these genomic profiles to predict long-term outcomes and potential response to emerging regimens, as discussed by Chen et al. (2021) [22], supports our interpretation that the “biological override” observed in this study reflects an active determinant of tumor behavior rather than a mere marker of clinical stage.

Our exploratory subgroup findings suggest that *KRAS* may be a principal contributor to the adverse survival signal observed in the pooled AGA cohort. This is consistent with prior literature linking *KRAS*-driven biology to aggressive behavior in biliary tract malignancies [23,24,25]. Notably, our findings suggest that the adverse effects of *KRAS* may be independent of co-occurring mutations or other clinical factors, a trend reinforced by Guangchao et al. (2026) [19], who highlighted the distinct prognostic weight of *KRAS* even in the presence of concurrent alterations [19]. By contrast, the *TP53* subgroup analysis was limited by a small sample size and should not be interpreted as evidence that *TP53* lacks prognostic relevance. While we describe these results as exploratory, explicit caution is recommended regarding their interpretation; these findings are primarily hypothesis-generating and require validation in larger, independent prospective cohorts. While grouping these distinct mutations under the umbrella of “actionable” alterations provides the statistical power necessary to analyze rare malignancies, we recognize that this monolithic approach is a simplification of a complex molecular landscape. Our subgroup data (Section 3.5) serves as an initial step toward parsing the distinct biological behaviors and prognostic weights of these individual drivers.

A notable finding of this study was the marked disparity in demographic documentation between cohorts before matching, particularly the substantially higher proportion of unknown race in the cohort without a documented AGA. This pattern likely reflects broader inequities in data completeness and access to molecular testing in routine oncology practice. Because GBC disproportionately affects certain geographic and ethnically enriched populations [20], unequal access to genomic evaluation may further widen disparities in precision oncology [26]. These observations support not only the broader use of molecular profiling in GBC but also more equitable implementation across healthcare settings.

**Table 3:** Comparative Genomic Landscape and Clinical Correlation.

Gene	Pathway/Function	Literature Correlation	Our Findings
*KRAS*	RAS/MAPK pathway Proliferation & metastasis	Jain et al. (2016) confirmed *KRAS* as a consistent adverse prognostic marker across global cohorts [27].	Primary driver of the “biological override” with a 13.1% absolute mortality increase.
*ERBB2 (HER2)*	Receptor tyrosine kinase Targetable subgroup and often co-occurs with PI3K/RAS alterations which may modulate therapeutic sensitivity and accelerate mortality.	Hu et al. (2025) documented significantly higher rates of *ERBB2* (*HER2*) alterations and female predominance in minority populations [28].	High female (76.5%) and minority representation (29.4% Asian/Hispanic) in this subgroup.
*IDH1*	Cellular metabolism; epigenetic regulation Mutations create oncometabolite 2-HG; define a distinct molecular subtype with potential metabolic vulnerabilities.	Borger et al. (2012) noted *IDH1* mutations are rare in primary GBC and more prevalent in intrahepatic cholangiocarcinoma [29].	Minimal cohort size (*N* = 10) prevented independent survival analysis.
*PIK3CA*	PI3K/AKT pathway activation Enhances proliferation, survival, and invasion; overlaps with *HER2* alterations	Giraldo et al. (2022) found *PIK3CA* mutations in 14% of GBC cases, frequently as a co-alteration with *ERBB2* [16].	Minimal cohort size (*N* = 10) prevented independent survival analysis.
*ARID1A*	SWI/SNF chromatin remodeling Loss-of-function associated with genomic instability and poor prognosis	Kang et al. (2022) described *ARID1A* loss as a driver of clonal expansion from precancerous lesions to GBC [24].	Minimal cohort size (*N* = 10) prevented independent survival analysis.
*TP53*	*DNA* damage response and apoptosis Most common tumor suppressor mutation	Li et al. (2014) identified *TP53* as the most frequent mutation (47%) in GBC [30]. Hu et al. (2025) [28] noted higher rates in African American patients. Nepal et al. (2021) found it in 29.3% of patients with GBC [31] and Brägelmann et al. (2021) found a 18% of genomic loses in Chilean patients with GBC [32]. Abdel-Wahab et al. (2020) found alterations of *TP53* in 61% of their cohort [8].	Second most common alteration (*N* = 34); associated with aggressive terminal phenotypes. Observed signal requires validation in larger independent cohorts.

Comparison of the current TriNetX Real-World Evidence (RWE) cohort findings with landmark genomic studies in gallbladder cancer (GBC). The “Literature Correlation” column identifies consistent and divergent trends between our massive federated database and historically smaller pathology-based or trial-based cohorts.

This study has several limitations. First, its retrospective design and reliance on EHR-derived data introduce the potential for residual confounding and coding misclassification. Second, while we matched for the occurrence of surgical resection and chemotherapy, we lacked granular detail on surgical quality, specifically, margin status (R0 vs. R1) and lymph node yield, both of which are primary determinants of survival in GBC. Third, the comparison cohort should not be interpreted as confirmed wild type, as it represents a “not documented” population, including patients who were never genomically profiled rather than patients with verified absence of AGAs. Fourth, TriNetX does not provide detailed information on assay platforms, panel breadth, variant pathogenicity, treatment sequencing, dose intensity, or consistent use of targeted therapies, all of which may influence survival. Finally, the mutation-specific subgroup analyses were exploratory and had variable statistical power.

Despite these limitations, this study provides large-scale, real-world evidence that the presence of documented AGAs is associated with poorer outcomes in GBC.

## 5. Conclusions

In this global real-world matched analysis, patients with gallbladder cancer and a documented AGA experienced higher mortality compared to those without documented alterations, even after matching for major demographic, stage-related, and treatment-related factors. These findings support both the potential prognostic significance of these genomic alterations in GBC and highlight the need to ensure equitable access to genomic testing and documentation. Prospective studies with mutation-specific annotation and treatment-level detail are needed to clarify the prognostic and therapeutic implications of individual alterations.

## Figures and Tables

**Table 1:** Cohort Characteristics After Propensity Score Matching (Model 1 vs. Model 2).

Characteristics	Model 1: Demographic Match (*N* = 659 Per Cohort) (Sensitivity Analysis)		Model 2: Oncologic Match (*N* = 381 per Cohort) (Primary Analysis)	
	No Documented AGA	≥1 Documented AGA	No Documented AGA	≥1 Documented AGA
Age (Mean SD)	70.4 (11.1)	70.2 (11.3)	69.8 (10.9)	69.7 (11.1)
Sex: Female (%)	58.9	59.0	61.2	61.0
Race: White (%)	67.2	67.1	68.5	68.5
Race: Black or AA (%)	15.6	15.3	14.8	14.8
Metastatic Status (%)	N/A	N/A	18.9	18.9
Surgical Resection (%)	N/A	N/A	24.7	24.7
Chemotherapy (%)	N/A	N/A	58.5	58.5
Max Standardized Mean Difference (SMD)	0.034		0.048	

Cohort 1 included patients without a documented AGA; Cohort 2 included patients with a documented AGA. Pre-propensity score-matched percentages are based on the *N* for each model. N/A: not applicable; oncologic variables were utilized exclusively for matching and analysis in the Model 2 primary cohort.

**Table 2:** Comparative Risk of All-Cause Mortality by Matching Strategy.

	Cohort	Patients (*N*)	Risk (%)	Risk Difference (%)	*p*-Value
Model 1	No Documented AGA	659	42.5	Reference	N/A
	≥1 Documented AGA	659	52.8	10.3	<0.001
Model 2	No Documented AGA	381	43.0	Reference	N/A
	≥1 Documented AGA	381	56.2	13.1	<0.001

Cumulative mortality risk was evaluated for patients with at least one documented actionable genomic alteration (AGA) versus those with no documented AGA. Model 1 (Demographic Match): 1:1 propensity score match for age, sex, and race/ethnicity. Model 2 (Oncologic Match): 1:1 propensity score match for age, sex, race/ethnicity, metastatic status, surgical resection, and chemotherapy history. Statistical Analysis: Risk Difference (RD) represents the absolute increase in mortality for the documented AGA cohort compared to the reference group. *p*-values were calculated using a two-sided z-test.

## Data Availability

The data presented in this study are available on request from the corresponding author. Restrictions apply to the availability of these data because they were obtained from the TriNetX Research Network under institutional access and data-use restrictions and are not publicly available. Researchers may obtain access directly from TriNetX, subject to its data access policies and institutional permissions.

## Supplementary Materials

The following supporting information can be downloaded at: https://www.mdpi.com/article/10.3390/cancers18091452/s1, Table S1: Clinical Characteristics by Genomic Subgroup.

## Abbreviations

The following abbreviations are used in this manuscript:

GBC	gallbladder cancer
AGA	actionable genomic alterations
